# Assessment of Hearing Screening Combined With Limited and Expanded Genetic Screening for Newborns in Nantong, China

**DOI:** 10.1001/jamanetworkopen.2021.25544

**Published:** 2021-09-17

**Authors:** Qing-Wen Zhu, Mu-Ting Li, Xun Zhuang, Kai Chen, Wan-Qing Xu, Yin-Hua Jiang, Gang Qin

**Affiliations:** 1 Clinical Medicine Research Center, Nantong Maternal and Child Health Hospital affiliated to Nantong University, Nantong, China; 2 Department of Epidemiology and Biostatistics, Nantong University School of Public Health, Nantong, China; 3 Department of Internal Medicine, Nantong University Medical School, Nantong, China

## Abstract

**Importance:**

Early identification and intervention for newborns with hearing loss (HL) may lead to improved physiological and social-emotional outcomes. The current newborn hearing screening is generally beneficial but improvements can be made.

**Objective:**

To assess feasibility and evaluate utility of a modified genetic and hearing screening program for newborn infants.

**Design, Setting, and Participants:**

This population-based cohort study used a 4-stage genetic and hearing screening program at 6 local hospitals in Nantong city, China. Participants were newborn infants born between January 2016 and June 2020 from the Han population. Statistical analysis was performed from April 1 to May 1, 2021.

**Exposures:**

Limited genetic screening for 15 variants in 4 common HL-associated genes and newborn hearing screening (NHS) were offered concurrently to all newborns. Hearing rescreening and/or diagnostic tests were provided for infants with evidence of HL on NHS or genetic variants on screening. Expanded genetic tests for a broader range of genes were targeted to infants with HL with negative results of limited genetic tests.

**Main Outcomes and Measures:**

The detection capability for infants with hearing impairment who passed conventional hearing screening, as well as infants with normal hearing at risk of late-onset HL due to genetic susceptibility.

**Results:**

Among a total of 35 930 infants, 32 512 infants completed the follow-up and were included for analysis. Among the infants included in the analysis, all were from the Han population in China and 52.3% (16 988) were male. The modified genetic and hearing screening program revealed 142 cases of HL and 1299 cases of genetic variation. The limited genetic screening helped identify 31 infants who passed newborn hearing screening, reducing time for diagnosis and intervention; 425 infants with normal hearing with pathogenic *SLC26A4* variation and 92 infants with *MT-RNR1* variation were at risk for enlarged vestibular aqueduct and aminoglycoside-induced ototoxicity respectively, indicating early aversive or preventive management.

**Conclusions and Relevance:**

This study found that performing modified genetic and hearing screening in newborns was feasible and provides evidence that the program could identify additional subgroups of infants who need early intervention. These findings suggest an advantage for universal adoption of such a practice.

## Key Points

**Question:**

Is the modified newborn genetic and hearing screening feasible in China?

**Findings:**

In this population-based cohort study including 32 512 infants, incorporating the limited and expanded genetic screening into physiological screening was associated with identifying 31 newborns with hearing loss missed by the conventional hearing screening, providing etiologic information to 1299 participants, and targeting 517 children at risk of late-onset hearing loss to improve prevention.

**Meaning:**

Large observational studies are needed to evaluate the cost-effectiveness and long-term benefits of integrated genetic and hearing screening programs.

## Teaser

This cohort study investigates the feasibility and utility of a modified genetic and hearing screening program among newborn infants in Nantong, China.

## Introduction

Hearing loss (HL) is the most common congenital sensory disorder, either as a solitary deficit (nonsyndromic HL or nsHL) or having other organs affected as well (syndromic HL or sHL).^1^ Approximately 50% to 60% of affected individuals have an identifiable genetic etiology.^2,3^ The prevalence of permanent childhood HL (PCHL) has been reported ranging from 0.3-15.0 per 1000 infants, with a median of 1.70.^4^ Missing the diagnosis of PCHL at an early stage may lead to lifelong impacts on the children, such as speech-language delay and both academic and social-emotional difficulties.^5^

Newborns with PCHL have benefitted from the universal newborn hearing screening (UNHS) program.^6^ However, newborn hearing screening is still unsatisfactory.^7^ On the one hand, it would miss newborns with less severe HL and would not identify late-onset prelingual HL.^8^ On the other hand, it would not elucidate the etiology that may indicate meaningful intervention.^2^ Among the genes identified to be causative for deafness, the following 4 have been extensively studied in the Chinese population^9,10,11,12^: *GJB2* gene encoding beta-2 gap junction protein, connexin 26; *SLC26A4* gene (causative for nonsyndromic HL or Pendred syndrome, formerly PDS gene) encoding pendrin; *MT-RNR1* gene encoding mitochondrial DNA 12s ribosomal RNA; and *GJB3* gene encoding beta-3 gap junction protein, connexin 31. Moreover, variants in *SLC26A4* and *MT-RNR1* are of particular interest, because they are responsible for HL associated with enlarged vestibular aqueduct (EVA) and aminoglycoside-induced ototoxicity, respectively. Identification of genetic carrier status provides an opportunity for reproductive counseling. Avoidance of potential event triggers (eg, intense physical exercise, aminoglycoside antibiotics) in individuals with such variants would dramatically reduce the incidence of HL.^13^

Limited genetic screening of a small number of HL-associated genes (*GJB2*, *SLC26A4*, and *MT-RNR1*) to improve the detection of late-onset prelingual HL was first proposed in 2006.^2^ Afterwards, concurrent limited genetic screening and hearing screening programs have been evaluated in several cities and provinces.^10,12,14,15,16,17,18^ The results show that limited genetic screening can identify newborns who otherwise might be missed by hearing screening alone and may identify individuals at risk for late-onset HL. Meanwhile, with the advent of next-generation sequencing technology, there has been an expansion in the number of deafness-related genes that can be screened, indicating the need for further evaluation.^19^

In Nantong, a city in east China adjacent to Shanghai, UNHS became mandated by the local authority in 2001 for use at all birthing hospitals.^6^ To identify hearing-impaired infants and those at risk of HL as early as possible, the Nantong government in 2014 recommended the implementation of a comprehensive newborn hearing and genetic screening program. We constructed a framework for integrating limited and expanded genetic testing into conventional newborn hearing screening. With the city-level model running for several years, we aimed to evaluate the program performance.

## Methods

### Study Design

This study used a concurrent newborn genetic and hearing screening program in Nantong city. From January 2016 to December 2020, the population-based cohort study was conducted at 6 hospitals: (1) Maternal and Child Health Hospital affiliated to Nantong University, (2) The Second Affiliated Hospital of Nantong University, (3) Nantong Second People’s Hospital, (4) Nantong Third People’s Hospital, (5) Nantong Sixth People’s Hospital, and (6) Nantong Rich Hospital. Race or ethnicity was collected by self-report at enrollment. All of the recruited infants received both newborn hearing screening and genetic screening for free, funded in part by the municipal government and research project foundations. This study followed the Strengthening the Reporting of Observational Studies in Epidemiology (STROBE) reporting guideline for cohort studies. This study was approved by the ethics committees of Nantong municipal Health Commission and all hospitals involved. Written informed consent was obtained from the infant’s parents.

Inclusion criteria were as follows: (1) the infants were born between January 2016 and June 2020 from the Han population in China; (2) the infants’ health condition was good enough to tolerate the screening procedures; (3) the parents were urban residents of Nantong city; and (4) the parents agreed to have their babies participating in the modified genetic and hearing screening program. Exclusion criteria were as follows: (1) the infants’ samples were unqualified for the genetic tests according to criteria of the National Health Commission of China’s technical specification for neonatal screening of congenital diseases; or (2) the infants were lost to hearing or genetic follow-up.

The population-based longitudinal databank for all children with congenital HL in Nantong city commenced in January 2016 and maintained indefinite recruitment and ongoing follow-up.

### Modified Genetic and Hearing Screening Program

The flowchart of the modified genetic and hearing screening program is illustrated in the Figure. Briefly, the work-up consisted of 4 stages: stage 1, at hospital before discharge (within 3 days), newborn hearing screening (NHS) and limited genetic screening tests were offered concurrently to the included infants; stage 2, at 42 days of age, hearing rescreening tests were offered to the infants with positive results of NHS and/or limited genetic screening; stage 3, at 3 months of age, diagnostic hearing tests were scheduled for the infants who did not pass hearing rescreening; stage 4, within 6 months, expanded genetic screening tests were conducted with the blood samples from those hearing-impaired patients with negative limited genetic screening results.

**Figure.**
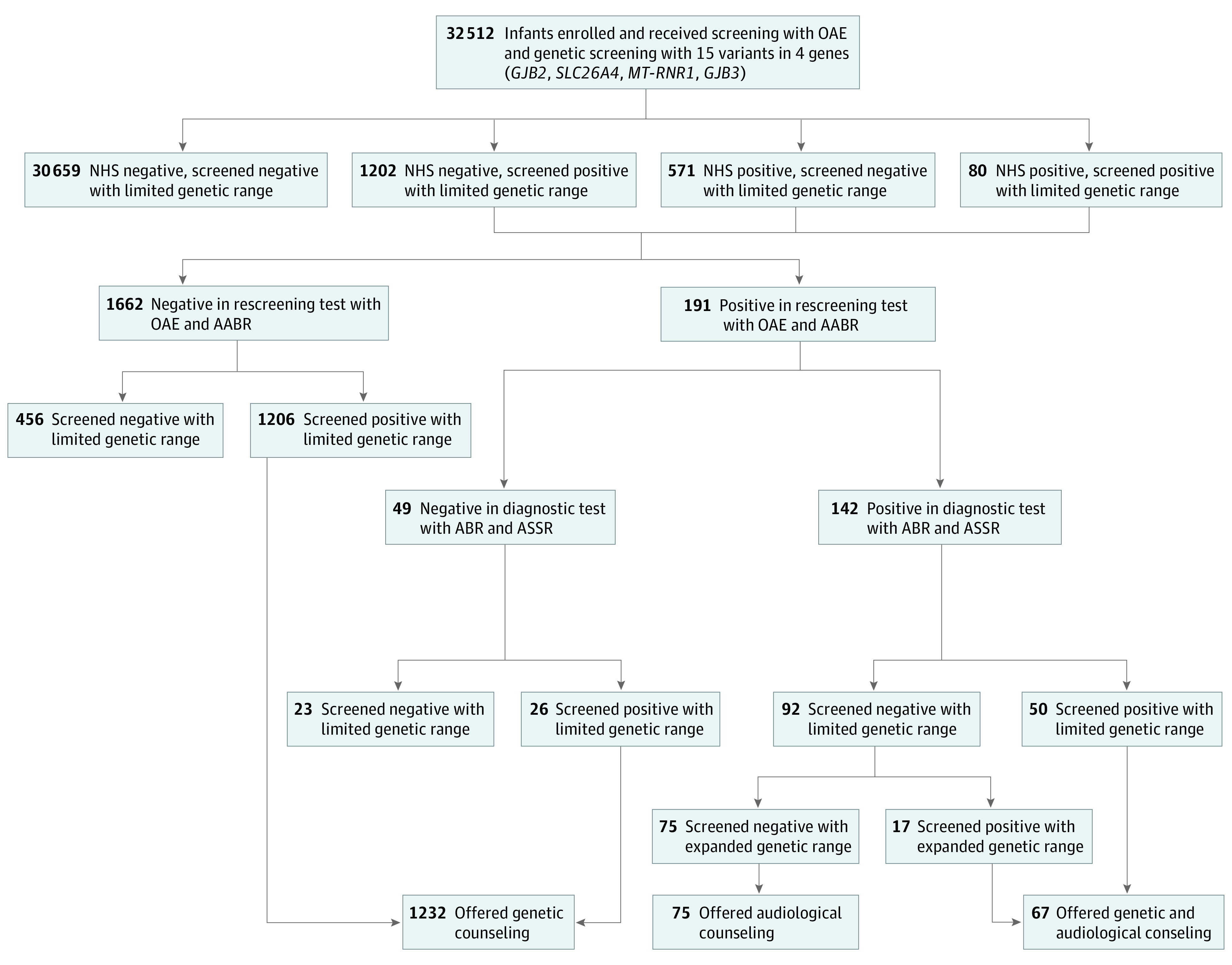
Overview and Results of the Modified Genetic and Hearing Screening Program AABR indicates automated auditory brainstem response; ABR, auditory brainstem response; ASSR, auditory steady state response; *GJB2*, gap junction beta-2; *GJB3*, gap junction beta-3; *MT-RNR1*, mitochondrial DNA12S-ribosomal RNA; OAE, otoacoustic emission; *SLC26A4*, solute carrier family 26, member 4.

Newborn hearing screening was conducted by otoacoustic emission (OAE). Hearing rescreening consisted of a repeat OAE and automated auditory brainstem response. Hearing diagnosis was made with auditory brainstem response and auditory steady state response. The severity of hearing loss was graded as mild (26-40 dB), moderate (41-60 dB), severe (61-80 dB), and profound (≥81 dB).^20^

Dried blood spot specimen was collected from the infants’ heel sticks. Genomic DNA was extracted by a blood filter paper nucleic acid extraction kit (CapitalBio, Beijing, China) and tested using a deafness gene variant detection array kit (CapitalBio, Beijing, China) with LuxScan 10K-B Microarray Scanner (CapitalBio, Beijing, China). A 2-step variation screening procedure, consisting of 4 genes (limited genetic screening for the general population), then 228 genes (expanded genetic screening for those who tested negative at the first step and confirmed HL) was performed. The limited genetic screening entailed genotyping 15 variants in 4 genes: c.35delG, c.176_191del16, c.235delC, c.299_300delAT (*GJB2* gene); c.1174A>T, c.1226G>A, c.1229C>T, c.1975G>C, c.2027T>A, c.2168A>G, c.IVS7-2A>G, c.IVS15 + 5G>A (*SLC26A4* gene); m.1494C>T, m.1555A>G (*MT-RNR1* gene); c.538C>T (*GJB3* gene). The results were categorized as (1) negative, (2) carrier (*GJB2* or *SLC26A4*, heterozygous mutations; *MT-RNR1* mutations; *GJB3* mutations; or heterozygous mutations in multiple genes), and (3) refer (*GJB2* or *SLC26A4*, homozygous or compound heterozygous mutations).^12^ The expanded genetic screening was conducted with next generation sequencing (NGS) panel (CapitalBio, Beijing, China) to identify 228 deafness-related genes by BioelectronSeq 4000 high throughput sequencing instrument (CapitalBio, Beijing, China) (eTable 1 in the Supplement). Variant interpretation was performed with the online platforms (ClinVar or Variant Interpretation Platform for Genetic Hearing Loss [VIP-HL]) according to the American College of Medical Genetics and Genomics and the Association for Molecular Pathology guidelines.^21^ In 2018 US dollars, the testing costs were $38 per individual for the limited genetic screening and $300 per individual for the expanded genetic screening.

The trained nurses offered general counseling to all newborns’ parents. The consulting included, but was not limited to, family history of HL, the workflow of the combined screening program, possible results, and referral arrangement. Elaborate counseling was offered to the parents in person if an infant did not pass either level of the screening. Audiological counseling, provided by audiologists, covered the clinical features and prognosis of HL, regular hearing monitoring, treatment options, rehabilitation with hearing aids or cochlear implants, and speech therapy. Genetic counseling, provided by genetic counselors, included the inheritance modes of hereditary HL, characteristics of nsHL and sHL, potential impact of positive genetic results on the family members, avoidance of trigger event exposure (for *SLC26A4* and *MT-RNR1* variant carriers), and routine hearing checkups (Figure).

### Statistical Analysis

All analyses were performed with Stata version 15.1 (StataCorp) from April 1 to May 1, 2021. χ^2^ tests were performed to determine the statistical significance of differences in groups. All reported *P* values were 2-tailed, and the significance threshold was defined as *P* < .05.

## Results

### Study Population

Between January 2016 and June 2020, a total of 35 930 infants born in Nantong were initially screened for the program. The health condition of 1426 infants did not allow newborn hearing screening tests within 3 days. The parents of 724 infants declined the genetic screening. For the 33 780 infants who underwent the modified genetic and hearing screening program, 986 blood samples were unqualified for genetic tests, 195, 81 and 6 infants lost to follow-up of newborn hearing screening, hearing rescreening and hearing diagnosis respectively. Overall, 32 512 infants who completed follow-up were included for analysis, of whom 52.3% (n = 16 988) were male, 47.7% (n = 15 524) were female, and all were from the Han population in China. The neonatal birth weight ranged from 2150 to 5100 g (mean [SD], 3312.6 [474.1] g). There were 1273 premature newborn infants (<37 weeks), accounting for 3.9%. Results of stages 1, 2, 3, and 4 of the concurrent genetic and hearing screening program were presented in eTable 2, eTable 3, eTable 4, and eTable 5 in the Supplement, respectively.

### Genotype and Phenotype Association of Variations

Among the cohort eligible for the analysis, the modified hearing and genetic screening program revealed 142 hearing loss cases and 1299 variants (Figure), resulting in an HL prevalence of 4.4 per 1000 infants (95% CI, 3.7-5.1 per 1000 infants) and a variation frequency of 4.0% (95% CI, 3.8%-4.2%) in our reference population. The variations in *GJB2* and *SLC26A4* were most enriched in the infants with HL with positive genotypes, accounting for 77.6% (52 of 67) of cases (Table 1).

**Table 1.** Genotype and Phenotype Association of 1299 Participants With Variations

Genotype	Participants, No.					
	Total	Hearing			Hearing loss	
		Normal	M/M	S/P	Confirmed diagnosis	Passed NHS
Limited genetic screening	1282	1232	17	33	50	31
Carrier	1260	1229	12	19	31	18
*GJB2* heter	666	651	3	12	15	11
*SLC26A4* heter	434	425	4	5	9	4
*MT-RNR1* heter/homo	87	86	1	0	1	1
*GJB3* heter/homo/CP	57	57	0	0	0	0
Multiple genes heter	16	10	4	2	6	2
Refer	22	3	5	14	19	13
*GJB2* homo/CP	15	2	4	9	13	9
*SLC26A4* homo/CP	7	1	1	5	6	4
Expanded genetic screening	17	0	5	12	17	NA
nsHL genes [*GJB2* homo/CP (3); *LOXHD1* CP (3); *TECTA* heter (2); *CCDC50* heter (1); *TRIOBP* CP (1)]	10	0	3	7	10	NA
sHL genes [*ADGRV1* CP (1); *CHD7* heter (1); *FGFR2* heter (1); *LRP2* CP (1); *USH2A* CP (1); *USH1G* homo with *PAX3* heter (1)]	6	0	2	4	6	NA
nsHL with sHL genes [*OTOG* CP with *MITF* heter (1)]	1	0	0	1	1	NA
Total	1299	1232	22	45	67	31

Abbreviations: *ADGRV1*, adhesion G protein-coupled receptor V1; *CCDC50*, coiled-coil domain containing 50; *CHD7*, chromodomain helicase DNA binding protein 7; CP, compound heterozygous in single gene; *FGFR2*, fibroblast growth factor receptor 2; *GJB2*, gap junction beta-2; *GJB3*, gap junction beta-3; heter, heterozygote; HL, hearing loss; homo, homozygote; *LOXHD1*, lipoxygenase homology domains 1; *LRP2*, low-density lipoprotein receptor-related protein 2; M/M, mild or moderate (26-60 dB); *MITF*, melanocyte inducing transcription factor; *MT-RNR1*, mitochondrial DNA12S-ribosomal RNA; NA, not applicable; NHS, newborn hearing screening; nsHL, nonsyndromic hearing loss; *OTOG*, otogelin; *PAX3*, paired box 3; S/P, severe or profound (≥61 dB); sHL, syndromic hearing loss; *SLC26A4*, solute carrier family 26, member 4; *TECTA*, tectorin alpha; *TRIOBP*, TRIO and F-actin binding protein; *USH1G*, Usher syndrome 1G; *USH2A*, Usher syndrome 2A.

Of 1260 participants in the carrier group, 31 (2.5%; 95% CI, 1.7%-3.5%) were reported to have HL, significantly higher than the population prevalence (142 of 32 512; 0.4% [95% CI, 0.3%-0.5%]; *P* < .001). However, none of the *GJB3 *variations was diagnosed with HL.

Among 22 participants in the refer group, 19 (86.4% [95% CI, 65.1%–97.1%]) were reported to have HL. The rates of HL in the *GJB2* positive and *SLC26A4* positive subgroups were similar (13 of 15 [86.7%] vs 6 of 7 [85.7%]; *P* = .95). Besides, both subgroups had predominantly severe to profound HL. Among 92 infants with HL passing limited genetic screening, expanded genetic screening further detected 17 infants (18.5%) with a variation (Table 1), including more nsHL genes (eg, *LOXHD1*, *TECTA*, and *CCDC50*) and sHL genes (eg, *ADGRV1*, *CHD7*, and *FGFR2*).

### Early Detection of HL Cases Missed by Conventional NHS

Of the 142 infants confirmed with HL at 3 months of age, 111 infants were diagnosed following the protocol of the conventional NHS program, whereas 31 infants (21.8% [95% CI, 15.3%-30.0%]) with variable degrees of HL passed NHS and were identified by the limited genetic screening, indicating early intervention (Table 2; eTable 6 in the Supplement). Among the 50 infants with HL identified with genetic variation, 62.0% (31 of 50) passed the newborn hearing screening. Moreover, it is worth noting that most of the missed cases (77.4%; 24 of 31) had severe to profound HL. Therefore, incorporating the genetic screening into newborn hearing screening helped to identify additional newborns with HL and to reduce time for diagnosis and intervention.

**Table 2.** Characteristics of Hearing Loss Cases Missed by the Conventional Newborn Hearing Screening

Gene	Variation	Classification of variants^a^	Participants, No.		
			Hearing loss		
			M/M	S/P	Total
*GJB2*	NM_004004.6:c.235delC heter	Pathogenic	1	8	9
	NM_004004.6:c.235delC homo	Pathogenic	0	4	4
	NM_004004.6:c.176_191del heter	Pathogenic	1	1	2
	NM_004004.6:c.35delG/ NM_004004.6:c.235delC CP	Pathogenic	1	1	2
	NM_004004.6:c.176_191del/ NM_004004.6:c.235delC CP	Pathogenic	0	2	2
	NM_004004.6:c.299_300delAT homo	Pathogenic	0	1	1
*SLC26A4*	NM_000441.2:c.919-2A>G heter	Pathogenic	2	2	4
	NM_000441.2:c.919-2A>G homo	Pathogenic	0	3	3
	NM_000441.2:c.2168A>G/ NM_000441.2:c.919-2A>G CP	Pathogenic	1	0	1
*MT-RNR1*	NC_012920.1:m.1494C>T homo	Drug response	1	0	1
Multiple genes heter	NM_004004.6(*GJB2*):c.235delC heter with NM_000441.2(*SLC26A4*):c.919-2A>G heter	Pathogenic	0	2	2
Total	NA	NA	7	24	31

Abbreviations: CP, compound heterozygous; *GJB2*, gap junction beta-2; heter, heterozygote; homo, homozygote; M/M, mild or moderate (26-60 dB); *MT-RNR1*, mitochondrial DNA12S-ribosomal RNA; NA, not applicable; S/P, severe or profound (≥61 dB); *SLC26A4*, solute carrier family 26, member 4.

^a^ Based on American College of Medical Genetics and Genomics (ACMG) guidelines for interpreting sequence variants.^18^

### Awareness and Precaution of HL Risk

The genetic and hearing screening program identified 425 of 32 512 infants (1.31%; 95% CI, 1.19%-1.44%) with *SLC26A4* variation who were at risk for EVA-associated HL (Table 3). For these children, anticipatory guidance was needed regarding the risk of external factors such as slapping, falls, and head injury. Besides, the screening program revealed 92 of 32 512 infants (0.28%; 95% CI, 0.23%-0.35%) carried with *MT-RNR1* variants and thus predisposed to ototoxicity. As 1 infant with mild to moderate HL reported no exposure to aminoglycoside drugs, the mitochondrial variant identified was unlikely the cause of HL in this case. The parents of mitochondrial variant carriers were informed about common aminoglycoside drugs and ototoxicity, and they were advised against such drug use for their children. Therefore, the genetic and hearing screening program enabled the identification of the targeted population (517 of 32 512; 1.59% [95% CI, 1.46%-1.73%]) that needs referral for special care and early aversive or preventive management.

**Table 3.** Normal-Hearing Carriers of *SLC26A4* and *MT-RNR1*

Gene	Variation	Classification of variant^a^	Cases, No.
*SLC26A4*	NM_000441.2:c.1174A>T heter	Pathogenic	3
	NM_000441.2:c.1226G>A heter	Pathogenic	16
	NM_000441.2:c.1229C>T heter	Pathogenic	11
	NM_000441.2:c.1975G>C heter	Pathogenic	5
	NM_000441.2:c.2027T>A heter	Pathogenic	1
	NM_000441.2:c.2168A>G heter	Pathogenic	59
	NM_000441.2:c.1707 + 5G>A heter	Pathogenic	2
	NM_000441.2:c.919-2A>G heter	Pathogenic	328
*MT-RNR1*	NC_012920.1:m.1494C>T heter	Drug response	3^b^
	NC_012920.1:m.1494C>T homo	Drug response	13
	NC_012920.1:m.1555A>G heter	Drug response	30^c^
	NC_012920.1:m.1555A>G homo	Drug response	46^d^
Total	NA	NA	517

Abbreviations: heter, heterozygote; homo, homozygote; *MT-RNR1*, mitochondrial DNA12S-ribosomal RNA; NA, not applicable; *SLC26A4*, solute carrier family 26, member 4.

^a^ Based on American College of Medical Genetics and Genomics (ACMG) guidelines for interpreting sequence variants.^18^

^b^ Including 1 case with mild or moderate (26-60 dB) hearing loss irrelevant to *MT-RNR1* mutation.

^c^ lncluding 1 case with multiple genes heterozygous mutation.

^d^ Including 4 cases with multiple genes heterozygous mutation.

### Childhood-Onset Hearing Loss During Follow-up

Except for the 142 infants with congenital HL, the follow-up system identified 11 cases of HL (9 male participants and 2 female participants) during the follow-up of past 5 years (Table 4). The median age at diagnosis for the participants was 10 months (interquartile range, 9-15 months). *GJB2* variation (3 cases), *SLC26A4* variation (1 case), and multiple genes heterozygous variation (5 cases) were involved in late-onset genetic HL in the population.

**Table 4.** Hearing Loss Cases Identified During the Follow-up of 2016-2020

Patient No.	Sex	Gene	Variation	Classification of variant^a^	Year/month of diagnosis	Age of diagnosis, mo	Grade of HL	Laterality
1	Male	Multiple genes heter	NM_004004.6(GJB2):c.299_300delAT heter with NM_000441.2(SLC26A4):c.919-2A>G heter	Pathogenic	2017/3	10	M/M	Bilateral
2	Female	*SLC26A4*	NM_000441.2:c.919-2A>G homo	Pathogenic	2018/3	7	S/P	Bilateral
3	Male	*GJB2*	NM_004004.6:c.299_300delAT homo	Pathogenic	2018/6	10	S/P	Bilateral
4	Male	*GJB2*	NM_004004.6:c.235delC heter	Pathogenic	2019/10	24	M/M	Bilateral
5	Male	Multiple genes heter	NM_004004.6(GJB2):c.235delC heter with NM_000441.2(SLC26A4):c.919-2A>G heter	Pathogenic	2019/4	16	S/P	Unilateral
6	Female	*GJB2*	NM_004004.6:c.235delC/ NM_004004.6:c.299_300delAT CP	Pathogenic	2019/5	15	S/P	Bilateral
7	Male	Negative	NA	NA	2019/7	15	M/M	Unilateral
8	Male	Multiple genes heter	NM_004004.6(GJB2):c.235delC heter with NM_000441.2(SLC26A4):c.919-2A>G heter	Pathogenic	2019/9	12	M/M	Bilateral
9	Male	Negative	NA	NA	2019/9	9	S/P	Unilateral
10	Male	Multiple genes heter	NM_004004.6(GJB2):c.299_300delAT heter with NM_000441.2(SLC26A4):c.919-2A>G heter	Pathogenic	2020/3	9	S/P	Bilateral
11^b^	Male	Multiple genes heter	NM_004004.6(GJB2):c.299_300delAT heter with NM_000441.2(SLC26A4):c.919-2A>G heter	Pathogenic	2020/3	9	S/P	Bilateral

Abbreviations: CP, compound heterozygous; *GJB2*, gap junction beta-2; heter, heterozygote; HL, hearing loss; homo, homozygote; M/M, mild or moderate (26-60 dB); NA, not applicable; S/P, severe or profound (≥61 dB); *SLC26A4*, solute carrier family 26, member 4.

^a^ Based on American College of Medical Genetics and Genomics (ACMG) guidelines for interpreting sequence variants.^18^

^b^ Twin brother to case 10.

## Discussion

Early identification and intervention for newborns with hearing loss was associated with improved physiological and social-emotional outcomes. The current newborn hearing screening is generally successful but improvements could be made. In the past decade, genetic testing has emerged as an important etiological diagnostic test for childhood HL. Some variants have been associated with environmental factors and thus regarded as preventable. Combined genetic and physiological screening may result in multiple benefits, including (1) identifying newborns with HL missed by the current physiological screen, (2) providing etiologic information, and (3) decreasing the number of children who would otherwise have late-onset HL. To our knowledge, we present a novel framework for integrating limited and expanded genetic testing into the current NHS and the performance assessment of the modified genetic and hearing program.

Among the 142 infants confirmed with HL at 3 months, we found that 21.8% (31/142; 95% CI, 15.3%-30.0%) passed the conventional newborn hearing screening. This is consistent with an earlier study that found the sensitivity of otoacoustic emission screening ranged from 79% for well babies and 96% for neonatal intensive care (NICU) graduates.^22^ OAE is considered to have low accuracy and lead to some false-negative errors.^23^ Besides, delayed onset of sensorineural HL limits our ability to achieve early identification of a substantial number of deaf infants.^7,24^ Of note, the median age at confirmation of permanent HL following a negative result of NHS was 25.7 months and 17.6 months for well babies and NICU graduates, respectively.^22^ The modified screening program identified not only babies born with HL but also those at risk for childhood-onset HL. Specialty counseling service to the parents would improve the hearing and language development of the HL children and protect those at risk.^15^

The genetic study of HL has greatly broadened our knowledge of normal auditory function and the pathophysiological processes that can disrupt it. HL-associated genes encoding different proteins involved in the development and function of the ear can affect any component of the hearing pathway.^25^ High prevalence of variation, especially of the 4 most common HL-associated genes, has been uncovered among the general population.^12,26^ Besides, an estimated 30% of genetic HL is syndromic. There are more than 100 genes associated with syndromic HL and more than 400 genetic syndromes that include HL as a feature, such as Pendred (EVA, thyroid goiter), Usher (retinitis pigmentosa), and Waardenburg (pigmentary anomalies) syndromes.^1^ Varying syndromic forms of HL may raise concerns about comorbidities that need specialty referral. Our study helps to understand the limits of limited genetic testing. When the result of limited genetic testing is negative, we have to be aware that HL would still have genetic etiology, because the variant associated with disease risk factors may be located in the region that has not been tested. The advantage to applying expanded genetic screening to this particular group is that targeted screening exhibits a high detection rate (18.5%, 17/92 in this study), favorably impacting clinical utility and cost-effectiveness.

If the newborn genetic and hearing screening is to be a priority, services for timely intervention must be established as well. Otherwise, early identification of HL provides no advantage. For congenital HL, hearing aids and cochlear implants are the primary treatments for the patients to improve hearing. Gene therapy, as a biological treatment, has the potential to restore hearing.^27,28,29^ However, there are still many safety concerns and translational hurdles to overcome before gene-editing technology can be used to treat HL.^30^ In our study, all the 153 HL infants have been referred to the specific hospitals and speech therapy service as well. Moreover, there has been no case of drug-induced HL during the follow-up period.

We should keep in mind that genetic screening has social and ethical implications. The 2 major concerns are (1) the privacy and confidentiality of genetic testing results and (2) the social and medical implications of any positive findings. Legislation and relevant policies should be available to protect individuals from discrimination by health insurers or employers based on genetic results.^31^

### Limitations

This study has several limitations. First, due to budget constraints, only an urban resident population was selected in the pilot study and observed for limited duration. Internal migrants (ie, floating population) were not included as the dropout rates among this subpopulation may be high.^32^ General population-based longitudinal studies are needed in the future to highlight the cost-effectiveness of this 4-stage screening strategy. Second, congenital cytomegalovirus (CMV) infection, the leading cause of nongenetic HL, was not included in this study. Although our previous decision model-based study suggested that screening for CMV may improve detection of newborns at risk for HL,^33^ we have not implemented the CMV screening project until early this year. Third, this study reported a surprisingly low incidence of childhood-onset HL during follow-up, considering difficulty of communication with families. The communication difficulties increased when the children grew older. Finally, this study was conducted in a relatively homogeneous Chinese population. It has been documented that etiologies (eg, genetic variations) of congenital HL might differ across different populations.^34,35^

## Conclusions

Today, approximately 30 Nantong infants are identified each year with congenital hearing loss within 3 months of birth, such that early intervention can be initiated to improve outcomes. Despite this, awareness and precaution of nearly 300 HL-associated gene variation carriers annually may bring further potential benefits. The results of our pilot study are expected to contribute to the knowledge concerning yield of the UNHS program. This study’s findings suggest that universal screening of a limited number of hotspot variants plus targeted screening for the hearing-impaired subpopulation would offer inexpensive and timely clinical benefits. The performance of Nantong's modified screening program highlights the need for universal adoption of such a practice. Our results call for rigorous large-scale observation to evaluate the cost-effectiveness and long-term benefits of integrated genetic and hearing screening programs.

## Notes

Supplement. **eTable 1.** Gene Panel For Next Generation Sequencing **eTable 2.** Results From Stage 1 of The Modified Genetic And Hearing Screening Program **eTable 3.** Results From Stage 2 of The Modified Genetic And Hearing Screening Program **eTable 4.** Results From Stage 3 of The Modified Genetic And Hearing Screening Program **eTable 5.** Results From Stage 4 of The Modified Genetic And Hearing Screening Program **eTable 6.** Characteristics of Hearing Loss Cases Missed by The Conventional NHS
eReference
