# Supplementary material for: Assessment of Hearing Screening Combined With Limited and Expanded Genetic Screening for Newborns in Nantong, China
Source: JAMA Netw Open. 2021 Sep 17;4(9):e2125544. doi: 10.1001/jamanetworkopen.2021.25544 (PMC8449278; doi:10.1001/jamanetworkopen.2021.25544)
Supplement: Supplement. — eTable 1. Gene Panel For Next Generation Sequencing eTable 2. Results From Stage 1 of The Modified Genetic And Hearing Screening Program eTable 3. Results From Stage 2 of The Modified Genetic And Hearing Screening Program eTable 4. Results From Stage 3 of The Modified Genetic And Hearing Screening Program eTable 5. Results From Stage 4 of The Modified Genetic And Hearing Screening Program eTable 6. Characteristics of Hearing Loss Cases Missed by The Conventional NHS eReference [file jamanetwopen-e2125544-s001.pdf]

Supplemental Online Content

Zhu QW, Li MT, Zhuang X, et al. Assessment of hearing screening combined with limited and expanded genetic screening for newborns in Nantong, China. *JAMA Netw Open.* 2021;4(9):e2125544.
doi:10.1001/jamanetworkopen.2021.25544

eTable 1. Gene Panel For Next Generation Sequencing

eTable 2. Results From Stage 1 of The Modified Genetic And Hearing Screening Program

eTable 3. Results From Stage 2 of The Modified Genetic And Hearing Screening Program

eTable 4. Results From Stage 3 of The Modified Genetic And Hearing Screening Program

eTable 5. Results From Stage 4 of The Modified Genetic And Hearing Screening Program

eTable 6. Characteristics of Hearing Loss Cases Missed by The Conventional NHS

eReference

This supplemental material has been provided by the authors to give readers additional information about their work.

© 2021 Zhu QW et al. *JAMA Network Open.*

eTable 1. Gene panel for next generation sequencing

**Non-syndromic HL genes**						
ACTG1	*ADCY1*	*AIFM1**	*ATP2B2*	*CABP2*	*CCDC50*	*CDC14A*
*CDH23**	*CEACAM16*	*CIB2*	*CLDN14*	*CLIC5*	*COCH*	*COL11A2**
COL4A6	*CRYM*	*DCDC2*	*DFNA5*	*DFNB59*	*DIABLO*	*DIAPH1*
*DSPP**	*ELMOD3*	*EPS8*	*ESPN*	*ESRRB*	*EYA4*	*FAM65B*
GIPC3	*GJB2*	*GJB3*	*GJB6*	*GPSM2*	*GRHL2*	*GRXCR1*
GRXCR2	*HGF*	*HOMER2*	*IFNLR1*	*ILDR1*	*KARS*	*KCNJ10**
KCNQ4	*KITLG*	*LHFPL5*	*LOXHD1*	*LRTOMT*	*MARVELD2*	*MET*
MSRB3	*MT-RNR1*	*MYH14*	*MYH9*	*MYO15A*	*MYO1A*	*MYO3A*
MYO6	*MYO7A**	*OSBPL2*	*OTOA*	*OTOF*	*OTOG*	*OTOGL*
P2RX2	*PCDH15**	*PNPT1*	*POU3F4*	*POU4F3*	*PRPS1*	*PTPRQ*
RDX	*S1PR2*	*SERPINB6*	*SIX1**	*SLC17A8*	*SLC26A4**	*SLC26A5*
SMPX	*STRC*	*SYNE4*	*TBC1D24**	*TECTA*	*TJP2*	*TMC1*
TMIE	*TMPRSS3*	*TNC*	*TPRN*	*TRIOBP*	*TSPEAR*	*USH1C**
*WFS1**	*WHRN*					
**Syndromic HL genes**						
ABCD1	*ABHD12*	*ADGRV1*	*ALMS1*	*ALX3*	*ALX4*	*ANKH*
ATP6V1B1	*ATP6V1B2*	*BCAP31*	*BCOR*	*BCS1L*	*BSND*	*C5ORF42*
CACNA1D	*CD151*	*CHD7*	*CHM*	*CISD2*	*CLCN7*	*CLCNKA*
CLCNKB	*CLPP*	*CLRN1*	*COL11A1*	*COL1A1*	*COL2A1*	*COL4A3*
COL4A4	*COL4A5*	*COL9A1*	*COL9A2*	*COLEC11*	*COQ6*	*DCAF17*
DHODH	*DIAPH3*	*DNAJC3*	*DNMT1*	*ECM1*	*EDN3*	*EDNRB*
ERCC3	*EYA1*	*FGF10*	*FGF3*	*FGFR1*	*FGFR2*	*FGFR3*
FLNA	*FOXC1*	*FOXI1*	*FRAS1*	*FREM2*	*GALE*	*GATA3*
GJA1	*GRIP1*	*HARS*	*HARS2*	*HMX1*	*HOXA2*	*HSD17B4*
IARS2	*IGF1*	*KCNE1*	*KCNQ1*	*LARS2*	*LHX3*	*LRP2*
LRP5	*MAF*	*MASP1*	*MED12*	*MGP*	*MITF*	*NDP*
NF1	*NF2*	*NLRP3*	*NOTCH2*	*OSTM1*	*PAX3*	*PDSS1*
PDZD7	*PEX1*	*PEX7*	*PHYH*	*PITX2*	*PLEKHM1*	*PLOD1*
PLOD3	*PMP22*	*POLD1*	*POLR1C*	*POLR1D*	*PRRX1*	*PTPN11*
RECQL4	*RMND1*	*SALL1*	*SALL4*	*SEMA3E*	*SERAC1*	*SF3B4*
SIX5	*SLC33A1*	*SLC4A11*	*SLITRK6*	*SNAI2*	*SNX10*	*SOST*
SOX10	*SOX9*	*SQSTM1*	*TBX1*	*TBX22*	*TCIRG1*	*TCOF1*
TCTN3	*TFAP2A*	*TGFB1*	*TIMM8A*	*TNFRSF11A*	*TNFRSF11B*	*TNFSF11*
TP63	*TRMU*	*TSHZ1*	*TWIST1*	*TWNK*	*TYR*	*USH1G*
USH2A	*ZNF687*					

*The genes were initially reported as non-syndromic HL genes but later found to cause certain syndromes.

Abbreviations: HL, hearing loss.

© 2021 Zhu QW et al. *JAMA Network Open.*

eTable 2. Results from stage 1 of the modified genetic and hearing screening program

**Limited genetic screening**	**Newborn hearing screening**		**Total**
	Pass	Fail	
Negative	30,659 (98.2%)	571 (1.8%)	31,230
Carrier	1,099 (94.1%)	69 (5.9%)*	1,168
Refer	103 (90.4%)	11 (9.6%)*	114
Total	31,861 (98.0%)	651 (2.0%)	32,512

Carrier group: heterozygous mutation of *GJB2* or *SLC26A4*, *MT-RNR1* mutation, *GJB3* mutation or multiple genes heterozygous mutation; Refer group: homozygous or compound heterozygous mutation of *GJB2* or *SLC26A4*.

*P < 0.001, compared with negative group.

© 2021 Zhu QW et al. *JAMA Network Open.*

eTable 3. Results from stage 2 of the modified genetic and hearing screening program

Limited genetic screening	Hearing re-screening		Total
	Pass	Fail	
Negative & NHS (+)	456 (79.9%)	115 (20.1%)	571
Carrier	1,114 (95.4%)	54 (4.6%)	1,168
Refer	92 (80.7%)	22 (19.3%)*	114
Total	1,662 (89.7%)	191 (10.3%)	1,853

Carrier group: heterozygous mutation of *GJB2* or *SLC26A4*, *MT-RNR1* mutation, *GJB3* mutation or multiple genes heterozygous mutation; Refer group: homozygous or compound heterozygous mutation of *GJB2* or *SLC26A4*.

Abbreviations: NHS, newborn hearing screening.

*P < 0.001, compared with the carrier group.

© 2021 Zhu QW et al. *JAMA Network Open.*

eTable 4. Results from stage 3 of the modified genetic and hearing screening program

Limited genetic screening	Classification of Variant[a]	Hearing diagnosis[b]			Total
		Normal	M/M	S/P	
**Carrier**					
GJB2					
NM_004004.6:c.176_191del heter	P	0	1	1	2
NM_004004.6:c.235delC heter	P	10	2	11	23
NM_004004.6:c.299_300delAT heter	P	1	0	0	1
SLC26A4					
NM_000441.2:c.1226G>A heter	P	0	0	1	1
NM_000441.2:c.2168A>G heter	P	1	0	0	1
NM_000441.2:c.919-2A>G heter	P	9	4	4	17
MT-RNR1					
NC_012920.1:m.1494C>T homo	drug response	1	1	0	2
Multiple genes heter					
NM_004004.6(*GJB2*):c.235delC hete with NM_000441.2(*SLC26A4*):c.919-2A>G hete	P	2	2	2	6
NM_004004.6(*GJB2*):c.299_300delAT hete with NM_000441.2(*SLC26A4*):c.919-2A>G hete	P	1	0	0	1
NM_000441.2(*SLC26A4*):c.919-2A>G hete with NM_024009.3(*GJB3*):c.538C>T hete	P	0	2	0	2
Sub-total		25	12	19	56
**Refer**					
GJB2					
NM_004004.6:c.235delC homo	P	0	1	4	5
NM_004004.6:c.299_300delAT homo	P	0	0	1	1
NM_004004.6:c.35delG/NM_004004.6:c.235delC CP	P	0	1	1	2
NM_004004.6:c.176_191del/NM_004004.6:c.235delC CP	P	0	1	2	3
NM_004004.6:c.176_191del/NM_004004.6:c.299_300delAT CP	P	0	1	0	1
NM_004004.6:c.235delC/NM_004004.6:c.299_300delAT CP	P	1	0	1	2
SLC26A4					
NM_000441.2(*SLC26A4*):c.919-2A>G homo	P	0	0	4	4
NM_000441.2:c.2168A>G/NM_000441.2:c.919-2A>G CP	P	0	1	1	2
Sub-total		1	5	14	20
**Total**		**26**	**17**	**33**	**76**

[a]Based on American College of Medical Genetics and Genomics (ACMG) guidelines for interpreting sequence variants.[1]
Abbreviations: *GJB2*, gap junction beta-2; *SLC26A4*, solute carrier family 26, member 4; *MT-RNR1*, mitochondrial DNA12S-ribosomal RNA; *GJB3*, gap junction beta-3; Hete, heterozygote; Homo, homozygous; CP, compound heterozygous; P, pathogenic; M/M, mild/moderate (26-60 dB); S/P, severe/profound (≥61 dB).

© 2021 Zhu QW et al. *JAMA Network Open.*

eTable 5. Results from stage 4 of the modified genetic and hearing screening program

Case	Sex	Grade of HL	Laterality	Gene	Genotype	Classification of variant[a]	Syndrome associated with the gene mutation	Syndrome characteristics
1	male	S/P	Bilateral	*CCDC50*	NM_178335.3:c.620C>G heter	VUS	nsHL	-
2	male	S/P	Bilateral	*LOXHD1*	NM_144612.6:c.2498G>A/ NM_144612.6:c.4283G>A CP	VUS	nsHL	-
3	male	S/P	Bilateral	*CHD7*	NM_017780.4:c.770A>G hete	VUS	CHARGE syndrome	Coloboma of the iris, retina, and/or optic disk; choanal atresia, cranial nerve dysfunction, characteristic CHARGE syndrome ear malformations, developmental delay, congenital cardiac defects, and poor growth.
4	female	S/P	Bilateral	*GJB2*	NM_004004.6:c.109G>A homo	P	nsHL	-
5	male	S/P	Bilateral	*FGFR2*	NM_000141.4:c.1216A>G hete	VUS	Apert syndrome	Premature fusion of cranial sutures with craniofacial deformities; digital deformities; mental retardation.
6	male	S/P	Bilateral	*LOXHD1*	NM_144612.6:c.163C>T/ NM_144612.6:c.1330C>G CP	VUS	nsHL	-
7	male	S/P	Bilateral	*LOXHD1*	NM_144612.6:c.163C>T/ NM_144612.6:c.1330C>G CP	VUS	nsHL	-
8	male	S/P	Bilateral	*LRP2*	NM_004525.3:c.2590G>A/ NM_004525.3:c.10629T>A CP	VUS	Donnai-barrow syndrome	Typical craniofacial features, vision and hearing loss，intellectual disability, agenesis of the corpus callosum, congenital diaphragmatic hernia, and omphalocele.

© 2021 Zhu QW et al. *JAMA Network Open.*

Case	Sex	Grade of HL	Laterality	Gene	Genotype	Classification of variant[a]	Syndrome associated with the gene mutation	Syndrome characteristics
9	female	S/P	Bilateral	*ADGRV1*	NM_032119.4:c.2330A>C/ NM_032119.4:c.4523A>G CP	VUS	Usher syndrome, type iic	Retinitis pigmentosa, and sensorineural hearing loss.
10	female	S/P	Bilateral	*TRIOBP*	NM_001039141.3:c.5266C>T/ NM_001039141.3:c.6862G>A CP	VUS	nsHL	-
11	male	S/P	Bilateral	*GJB2*	NM_004004.6:c.109G>A/ NM_004004.6:c.235delC CP	P	nsHL	-
12	male	S/P	Bilateral	Multiple genes heter	NM_001277269.2(*OTOG*):c.388C>G/ NM_001277269.2(*OTOG*):c.6562A>C CP with NM_001354604.2(*MITF*):c.658A>G hete	VUS	nsHL (*OTOG*) / Waardenburg syndrome, type 2a (*MITF*)	Sensorineuronal hearing loss and pigmentary disturbance (*MITF*).
13	female	M/M	Bilateral	Multiple genes heter	NM_173477.5(*USH1G*):c.164+5G>A homo with NM_181458.4(*PAX3*):c.339C>G hete	VUS	Usher syndrome, type ig (*USH1G*) / Waardenburg syndrome, type 1 (*PAX3*)	Onset of retinitis pigmentosa by 10 years (*USH1G*) / Craniofacial dysmorphism with dystopia canthorum; pigmentation abnormalities (*PAX3*).
14	male	M/M	Bilateral	*GJB2*	NM_004004.6:c.109G>A/NM_004004.6:c. 571T>C CP	P	nsHL	-
15	male	M/M	Bilateral	*TECTA*	NM_005422.2:c.859G>A hete	VUS	nsHL	-

© 2021 Zhu QW et al. *JAMA Network Open.*

Case	Sex	Grade of HL	Laterality	Gene	Genotype	Classification of variant[a]	Syndrome associated with the gene mutation	Syndrome characteristics
16	female	M/M	Unilateral	*TECTA*	NM_005422.2:c.3043G>A hete	VUS	nsHL	-
17	female	M/M	Unilateral	*USH2A*	NM_206933.3:c.5608C>T/ NM_206933.3:c.8603A>G CP	VUS	Usher syndrome, type iia	Onset of retinitis pigmentosa in late teens/ early adulthood.

[a]Based on American College of Medical Genetics and Genomics (ACMG) guidelines for interpreting sequence variants.[1]

Abbreviations: M/M, mild/moderate (26-60 decibel); S/P, severe/profound (≥61 decibel); Heter, heterozygote; Homo, homozygous; CP, compound heterozygous; P, pathogenic; VUS, variant of uncertain significance; HL, hearing loss; nsHL, non-syndromic HL; *GJB2*, gap junction beta-2; *LOXHD1*, lipoxygenase homology domains 1; *TECTA*, tectorin alpha; *CCDC50*, coiled-coil domain containing 50; *TRIOBP*, TRIO and F-actin binding protein; *ADGRV1*, adhesion G protein-coupled receptor V1; *CHD7*, chromodomain helicase DNA binding protein 7; *FGFR2*, fibroblast growth factor receptor 2; *LRP2*, low-density lipoprotein receptor-related protein 2; *USH2A*, Usher syndrome 2A; *USH1G*, Usher syndrome 1G; *PAX3*, paired box 3; *OTOG*, otogelin; *MITF*, melanocyte inducing transcription factor.

© 2021 Zhu QW et al. *JAMA Network Open.*

eTable 6. Characteristics of hearing loss cases missed by the conventional NHS

Case	Sex	Gene	Variation	Classification of variant[a]	Grade of HL	Laterality
**Carrier**						
1	female	*GJB2*	NM_004004.6:c.235delC heter	P	S/P	bilateral
2	male	*GJB2*	NM_004004.6:c.235delC heter	P	S/P	unilateral
3	female	*GJB2*	NM_004004.6:c.235delC heter	P	S/P	unilateral
4	male	*SLC26A4*	NM_000441.2:c.919-2A>G heter	P	M/M	unilateral
5	male	*GJB2*	NM_004004.6:c.235delC heter	P	M/M	unilateral
6	male	Multiple heterozygous[b]	NM_004004.6(*GJB2*):c.235delC with NM_000441.2(*SLC26A4*):c.919-2A>G heter	P	S/P	unilateral
7	male	*SLC26A4*	NM_000441.2:c.919-2A>G heter	P	M/M	bilateral
8	male	*GJB2*	NM_004004.6:c.176_191del heter	P	M/M	unilateral
9	male	*SLC26A4*	NM_000441.2:c.919-2A>G heter	P	S/P	unilateral
10	male	*SLC26A4*	NM_000441.2:c.919-2A>G heter	P	S/P	unilateral
11	female	*GJB2*	NM_004004.6:c.176_191del heter	P	S/P	unilateral
12	male	*GJB2*	NM_004004.6:c.235delC heter	P	S/P	unilateral
13	male	*GJB2*	NM_004004.6:c.235delC heter	P	S/P	unilateral
14	male	*GJB2*	NM_004004.6:c.235delC heter	P	S/P	unilateral
15	female	*GJB2*	NM_004004.6:c.235delC heter	P	S/P	unilateral
16	male	Multiple heterozygous[b]	NM_004004.6(GJB2):c.235delC heter with NM_000441.2(*SLC26A4*):c.919-2A>G heter	P	S/P	bilateral
17	female	*GJB2*	NM_004004.6:c.235delC heter	P	S/P	unilateral
**Refer**						
1	male	*GJB2*	NM_004004.6:c.176_191del/ NM_004004.6:c.235delC CP	P	S/P	unilateral
2	male	*SLC26A4*	NM_000441.2:c.2168A>G/ NM_000441.2:c.919-2A>G CP	P	M/M	bilateral
3	male	*GJB2*	NM_004004.6:c.235delC homo	P	S/P	bilateral
4	male	*SLC26A4*	NM_000441.2:c.919-2A>G homo	P	S/P	bilateral
5	female	*SLC26A4*	NM_000441.2:c.919-2A>G homo	P	S/P	bilateral
6	male	*GJB2*	NM_004004.6:c.299_300delAT homo	P	S/P	bilateral
7	female	*GJB2*	NM_004004.6:c.176_191del/ NM_004004.6:c.235delC CP	P	S/P	bilateral
8	male	*SLC26A4*	NM_000441.2:c.919-2A>G homo	P	S/P	bilateral
9	female	*GJB2*	NM_004004.6:c.235delC homo	P	S/P	bilateral
10	female	*GJB2*	NM_004004.6:c.235delC homo	P	S/P	bilateral
11	female	*GJB2*	NM_004004.6:c.235delC homo	P	S/P	bilateral
12	male	*GJB2*	NM_004004.6:c.35delG/ NM_004004.6:c.235delC CP	P	M/M	unilateral
13	male	*GJB2*	NM_004004.6:c.35delG/	P	S/P	bilateral

© 2021 Zhu QW et al. *JAMA Network Open.*

			NM_004004.6:c.235delC CP			
14	female	*MT-RNR1*	NC_012920.1:m.1494C>T homo	drug response	M/M	bilateral

[a]Based on American College of Medical Genetics and Genomics (ACMG) guidelines for interpreting sequence variants.[1]
[b]Not only one type of gene with mutation was multiple heterozygous.
Abbreviations: NHS, newborn hearing screening; *GJB2*, gap junction beta-2; *SLC26A4*, solute carrier family 26, member 4; *MT-RNR1*, mitochondrial DNA12S-ribosomal RNA; Heter, heterozygote; Homo, homozygote; CP, compound heterozygous; P, pathogenic; M/M, mild/moderate (26-60 decibel); S/P, severe/profound (≥61 dB).

© 2021 Zhu QW et al. *JAMA Network Open.*

eReference

1. Richards S, Aziz N, Bale S, et al. Standards and guidelines for the interpretation of sequence variants: a joint consensus recommendation of the American College of Medical Genetics and Genomics and the Association for Molecular Pathology. *Genet Med.* 2015;17(5):405-424.

© 2021 Zhu QW et al. *JAMA Network Open.*
